# Tissue remodeling after RNAi-mediated knockdown of TTR in a Familial Amyloidotic Polyneuropathy mouse model

**DOI:** 10.1186/1750-1172-10-S1-P5

**Published:** 2015-11-02

**Authors:** Nádia Pereira Gonçalves, Paula Gonçalves, Miguel Ventosa, Ana Varela Coelho, Maria João Saraiva

**Affiliations:** 1 Instituto de Inovação e Investigação em Saúde (I3S), Instituto de Biologia Molecular e Celular (IBMC), Universidade do Porto, Molecular Neurobiology Unit, 4150-180, Porto, Portugal; 2 Instituto de Tecnologia Química e Biológica António Xavier (ITQB), Universidade Nova de Lisboa, Mass Spectrometry, 2781-901, Oeiras, Portugal

## Background

Transthyretin (TTR) deposition in the peripheral nervous system (PNS) is the hallmark of Familial Amyloidotic Polyneuropathy (FAP). Mice expressing human TTR with the V30M mutation in a heterozygous heat shock factor 1 (Hsf-1) background show extensive TTR deposits in PNS and gastrointestinal tract, as well as extracellular matrix (ECM) remodeling, similar to those seen in human FAP patients. Currently, liver transplantation is the only available treatment to halt the progression of clinical symptoms in FAP. Due to the limitations of this procedure, it is of utmost importance to develop alternative therapeutic strategies. In this regard, an RNAi therapeutic targeting TTR for the treatment of FAP is currently in Phase 3 clinical development. An ongoing phase 2 clinical trial in FAP patients demonstrated promising results as a mean plasma TTR reduction of 80%, sustained for over nine months, led to a decrease in neuropathy progression compared to historical data.

## Methods

To dissect molecular changes occurring in tissues upon RNAi-mediated knockdown of TTR, we treated both chronically and acutely the Hsf/V30M FAP mouse model, in different stages of TTR deposition and analyzed histopathological and biochemical changes in the most affected organs.

## Results

Our data show that inhibition of TTR expression by the liver prevent and reverse TTR deposition in PNS and GI tract. In addition, this treatment resulted in ECM remodeling with decreased levels of matrix metalloproteinase-2 (MMP-2) expression and MMP-9 activity in dorsal root ganglia. Importantly, MMP-2 protein levels were found down regulated in plasma samples from older mice treated with RNAi while animals treated with Tafamidis, Anakinra or Doxycycline/TUDCA showed no difference, suggesting that ECM remodeling with decreased MMP-2 might be a specific effect of RNAi.

## Conclusion

Collectively, our data show that silencing TTR liver synthesis in vivo can modulate TTR-induced pathology in the PNS.

